# An international survey of diffusion and perfusion magnetic resonance imaging implementation in the head and neck

**DOI:** 10.1007/s00330-025-11370-1

**Published:** 2025-02-04

**Authors:** Steve Connor, Alexander Christoforou, Philip Touska, Soraya Robinson, Nancy J. Fischbein, Pim de Graaf, Anne R. J. Péporté, Jussi Hirvonen, Darka Hadnadjev Šimonji, Gloria J. Guzmán Pérez-Carrillo, Xin (Cynthia) Wu, Christine Glastonbury, Kristine M. Mosier, Ashok Srinivasan

**Affiliations:** 1 School of Biomedical Engineering and Imaging Sciences, King’s College London, London, UK; 2 Department of Neuroradiology, King’s College Hospital, London, UK; 3 Department of Radiology, Guy’s Hospital and St Thomas’ Hospital, London, UK; 4 King’s College London, Guy’s Campus, London, UK; 5 Imaging Urania, Vienna, Austria; 6 Division of Neuroimaging and Neurointervention, Department of Radiology, Stanford University Medical Center, Stanford, CA USA; 7 7Department of Radiology and Nuclear Medicine, Amsterdam UMC, Vrije Universiteit, Amsterdam, The Netherlands; 8 Cancer Center Amsterdam, Imaging and Biomarkers, Amsterdam, The Netherlands; 9 Department of Radiology, Cantonal Hospital, Frauenfeld, Switzerland; 10 Department of Radiology, Tampere University Hospital, Tampere, Finland; 11 Faculty of Medicine and Health Technology, University of Tampere, Tampere, Finland; 12 Center for Radiology, Clinical Center of Vojvodina, Novi Sad, Serbia; 13 Faculty of Medicine, University in Novi Sad, Novi Sad, Serbia; 14 Mallinckrodt Institute of Radiology, Washington University School of Medicine, St. Louis, MO USA; 15 Department of Radiology and Biomedical Imaging, University of California, San Francisco, USA; 16 Department of Radiology and Imaging Sciences, Indiana University School of Medicine, Indianapolis, IN USA; 17 Division of Neuroradiology, Department of Radiology, University of Michigan, Ann Arbor, MI USA

**Keywords:** Neck, Magnetic resonance imaging, Perfusion imaging, Diffusion imaging, Surveys and questionnaires

## Abstract

**Objective:**

The goal of this international survey was to understand how diffusion (DWI) and perfusion imaging (PWI) are being applied to clinical head and neck imaging.

**Methods and materials:**

An online questionnaire focusing on acquisition, clinical indications, analysis, and reporting of qualitative DWI (QlDWI), quantitative DWI (QnDWI) and dynamic contrast-enhanced PWI (DCE-PWI) in the head and neck was circulated to members of the American Society of Head and Neck Radiology (ASHNR) and European Society of Head and Neck Radiology (ESHNR) over a 3-month period. Descriptive statistics and group comparisons were calculated with SPSS® v27.

**Results:**

There were 294 unique respondents (17.6% response rate) from 256 institutions (182 ESHNR, 74 ASHNR). DWI was routinely acquired for some head and neck indications at 95.7% of the respondents’ institutions, with 92.5% of radiologists interpreting QlDWI but only 36.7% analysing QnDWI. QlDWI was most frequently applied to primary mucosal masses or the middle ear, whilst QnDWI was routinely used to distinguish tumour histologies, and primary or recurrent carcinoma. DCE-PWI was routinely acquired at 53.6% of institutions and used by 40.8% of respondents, however, there was no clinical scenario in which it was routinely applied by most users. DCE-PWI analysis methods varied, with time-intensity curve classifications being the most frequently reported. Lack of standardisation was identified as a key reason for not implementing QnDWI, whilst numerous factors prevented the adoption of DCE-PWI.

**Conclusion:**

There is widespread routine interpretation of QlDWI by head and neck radiologists, but there is considerable variation in the application and analysis of head and neck QnDWI and DCE-PWI.

**Key Points:**

***Question*** *How are diffusion (DWI) and dynamic contrast-enhanced perfusion imaging (DCE-PWI) being utilised by head and neck radiologists across a wide range of practices*?

***Findings*** *An international survey demonstrated widespread routine interpretation of qualitative DWI but variable application and analysis of quantitative DWI and DCE-PWI with numerous barriers to implementation*.

***Clinical relevance*** *The survey results will aid discussion on how to standardise and optimally disseminate these MRI techniques in day-to-day practice. More focused education and resource allocation may be required to accelerate the adoption of quantitative DWI and DCE-PWI*.

## Introduction

Diffusion-weighted imaging (DWI) and perfusion-weighted imaging (PWI) are advanced imaging techniques that may be used to supplement conventional magnetic resonance imaging sequences in the head and neck. DWI is a fast, readily available sequence which depicts molecular diffusivity and provides information on tissue microstructure [1]. Whilst it may be reported qualitatively, it also offers a quantitative metric, the apparent diffusion coefficient (ADC) that is reflective of the freedom of water molecular movement [2–6]. DWI is often used to evaluate cellular tumours and other head and neck lesions with restricted diffusion such as purulent fluid or cholesteatoma. Dynamic contrast-enhanced (DCE) imaging is the most frequently applied method for evaluating perfusion with head and neck MRI, although non-contrast PWI methods such as arterial spin labelling are also available [7]. DCE-PWI is a more challenging sequence to acquire and analyse than DWI [8–10]. It evaluates microcirculatory tissue characteristics, thereby providing information on tumour neo-angiogenesis or tissue hypoxia and differentiating lesions based on their vascular architecture. PWI may be evaluated on a qualitative basis wherein simple semi-quantitative methods will calculate parameters from the time intensity curve. Alternatively, a more complex quantitative analysis can provide an array of more physiologically meaningful metrics.

DWI and DCE-PWI have a wide range of potential applications in the head and neck, ranging from diagnosis, delineation and monitoring of treatment response [11–16] in malignant neoplasms to the characterisation of benign disease [17–21]. The utilisation of these techniques is regularly incorporated in educational lecture programmes and publications [22–25] and has been the subject of both systematic reviews [12, 16, 18, 19, 26–31], and practice recommendations [32–35]. Existing literature demonstrates that DWI is often routinely incorporated into head and neck imaging protocols whilst DCE-PWI is increasingly translated from the research to the clinical domain. Variable utilisation may nonetheless result from a lack of resources, time pressures, technical challenges, insufficient knowledge or evidence, and inadequate standardisation of these techniques. In addition, as there is no universally accepted approach to the measuring and reporting of functional parameters [33], it remains unclear which methods are being used in clinical work.

The aim of this structured survey was to help us better understand when and how DWI and DCE-PWI are being utilised and practised by head and neck radiologists internationally, and whether there are barriers to their implementation. Improved understanding will be an important step towards increasing awareness of these techniques and supporting their consistent adoption in day-to-day practice.

## Method

### Questionnaire formulation

This study was based on a survey of head and neck imaging specialists with no patient data, so Institutional Review Board approval was not required. A draft questionnaire was formulated by the lead author (S.C.) which focused on the clinical application, analysis, and reporting of qualitative DWI (QlDWI), quantitative DWI (QnDWI) and DCE-PWI in the head and neck. The survey also included questions about potential barriers to implementation and intentions to apply these MRI techniques in the future.

A peer group of 12 head and neck subspecialty radiologists from the American Society of Head and Neck Radiology (ASHNR) and European Society of Head and Neck Radiology (ESHNR) provided content validation through an iterative review process and mutual agreement. The online version of the questionnaire was implemented with Microsoft Forms (Microsoft Corp) (International survey of current practice in diffusion and perfusion imaging of head and neck). A pilot test was performed within the institutions of the peer group members.

Allowing for the use of branching questions, the survey required up to 25 responses and took approximately 5 min to complete (Supplementary Fig. S1). Questions referred to institutional acquisition of DWI and DCE or arterial spin labelling (ASL) PWI and individual practice in analysing QlDWI, QnDWI, and DCE-PWI, as well as the most applicable clinical indications and anatomical sites. Questions also probed how QnDWI and DCE-PWI were analysed and reported, organisational aspects of requesting and post-processing DCE-PWI, barriers preventing their implementation, and intentions to adopt these techniques in the head and neck. Where responses were optional, participants could choose to omit sections according to their level of certainty. Mandatory contact e-mail and practice details allowed the identification of multiple responses from a single institution.

### Questionnaire distribution

The questionnaire was distributed by e-mail to the membership of the ESHNR and ASHNR with an explanatory text. The e-mail invitations were coordinated by the two societies and were sent out on the first week of March, mid-April, and mid-May, with the questionnaire open to responses from March 1 to May 31, 2024. All responses were treated confidentially.

### Analysis of responses

Responses were collated within the online survey site and within Microsoft Excel. Descriptive statistics and group differences were analysed with SPSS® v27.0 (IBM Corp.). Duplicated responses or redundant blank forms were screened and removed.

Proportions were reported as percentages with 95% confidence intervals (95% CI), with the denominator being respondents or institutions depending on the analysis. Mean and standard deviation were reported for absolute numbers when normally distributed (Shapiro–Wilk).

Group comparisons with Chi-squared were performed to explore associations between the routine acquisition of DWI and DCE-PWI and whether the institution was based in Europe versus North America, the number of head and neck radiologists reporting MRI (< 0–3, 4–7, and > 7), and whether it was a public, academic or private practice institution. The proportions of European radiologists and North American radiologists utilising QlDWI, QnDWI and DCE-DWI utilisation were also compared. A *p* value < 0.05 was considered statistically significant.

## Results

### Demographics

**Fig. 1:**
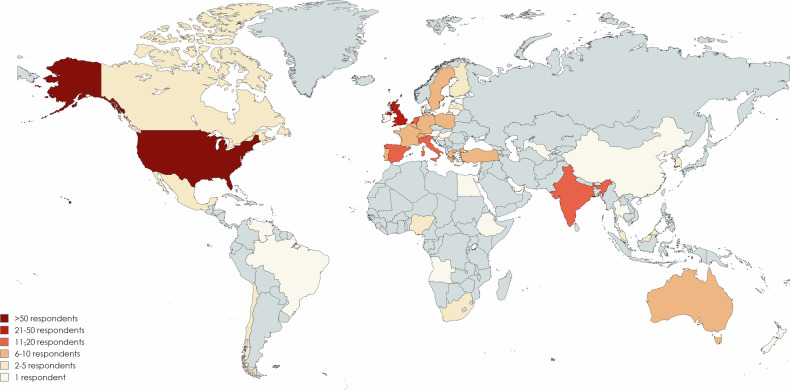
World map indicating the number of respondents from each country. A magnified map showing the number of responses from each European country is shown in Supplementary Fig. 1. Note the society membership spreads beyond Europe and North America although group comparisons were confined to those working in Europe and North America alone

**Fig. 2:**
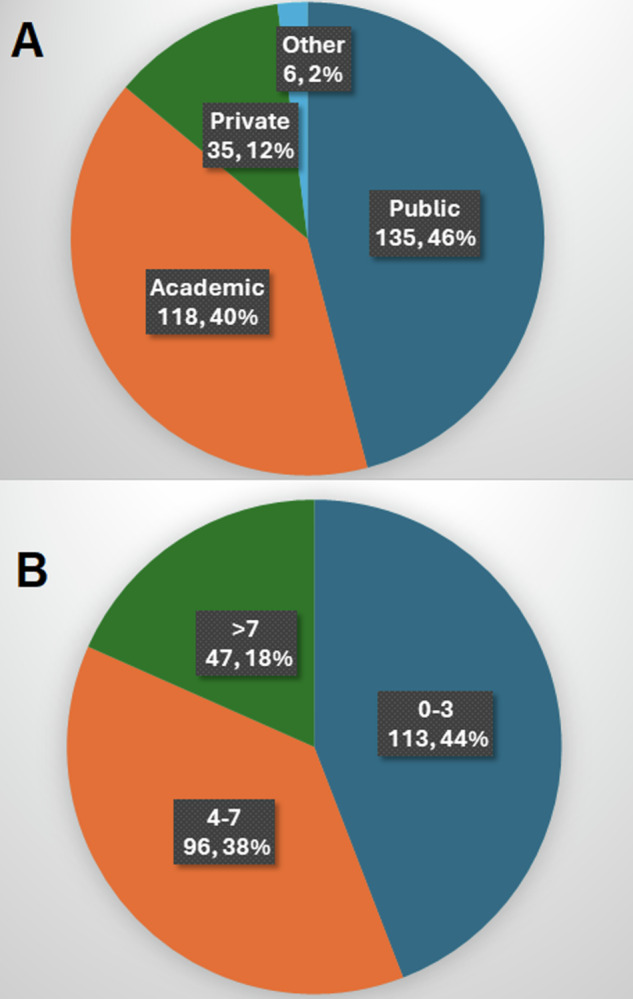
Pie charts indicate (**A**) the number and proportion of total respondents (*n* = 294) based on public institutions, academic institutions and private practice institutions. Since there could be overlap in some of these categories where an institution could be both public and academic, the respondents were asked to pick the best possible single option based on their work profiles. **B** The number of post-certification radiologists regularly reporting head and neck MRI in each institution (0–3, 4–7, and > 7)

The survey was e-mailed to a maximum of 822 ESHNR and 850 ASHNR members, with 209 (25.4%) and 85 (10%) unique respondents respectively. There was, therefore, a total of 294 respondents who practised in 44 different countries (Fig. 1, Supplementary Fig. S2, and Supplementary Table S1). Respondents were primarily based in 256 different institutions (ESHNR *n* = 182; ASHNR *n* = 74) which were most frequently located in the United Kingdom (*n* = 41) and the United States of America (*n* = 57). Most respondents were primarily based in public institutions (135/294; 45.9%; 95% CI: 40.1–51.8%) and academic institutions (118/294; 40.1%; 95% CI: 34.5–45.6%) with only 35/294 (11.9%; 95% CI: 8.4–16.2%) primarily based in private practice institutions. The number of board-certified, attending or consultant radiologists regularly reporting head and neck MRI in each institution were 1–3 in 113/256 (44.1%; 95% CI: 38.0–50.5%), 4–7 in 96/256 (37.5%; 95% CI: 31.5–43.7%) and > 7 in 47/256 (18.4%; 95% CI: 13.8–23.7%) (Fig. 2).

### Institutional acquisition and individual utilisation of QlDWI, QnDWI, and DCE-PWI

**Fig. 3:**
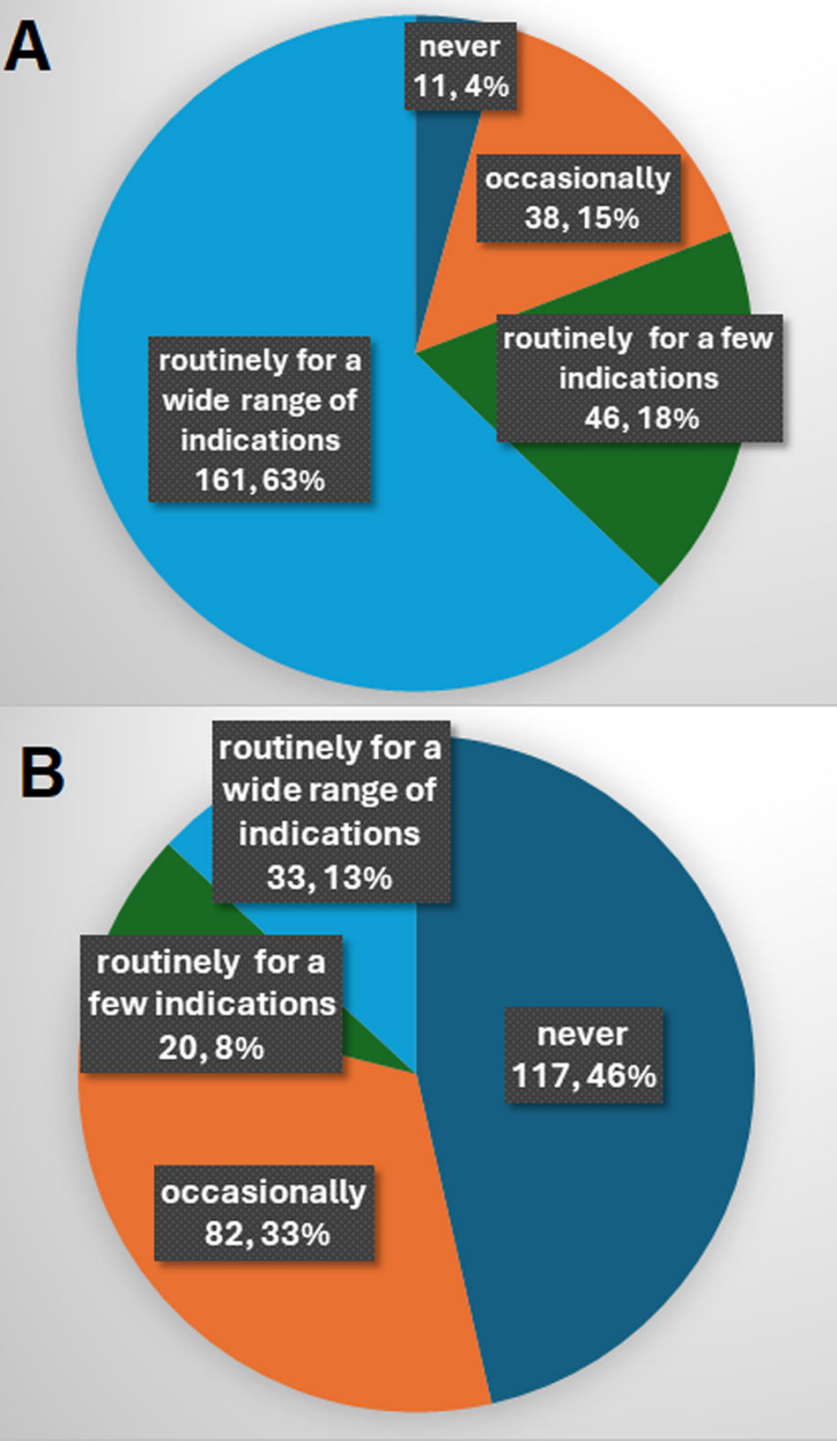
Pie charts indicating the number and proportion of all institutions (*n* = 256) where (**A**) DWI and (**B**) DCE-PWI are never acquired, occasionally acquired, routinely acquired for a narrow range of indications or routinely acquired for a wide range of indications

DWI in the head and neck was acquired at 245 of 256 (95.7%; 95% CI: 92.4–97.8%) institutions and was performed routinely for a wide range of indications at 161/256 (62.9%; 95% CI: 56.7–68.8%) (Fig. 3). Overall, 272 of 294 (92.5%; 95% CI: 88.9–95.3%) respondents interpreted QlDWI and 108 of 294 (36.7%; 95% CI: 31.2–42.5%) analysed QnDWI (Table 1 and Supplementary Fig. S3)

**Table 1:** Respondents interpreting QlDWI, analysing QnDWI and using DCE-PWI or ASL-PWI

	*n* = 294
QlDWI (interpret DWI signal and/or ADC maps)	272 (92.5%)
QnDWI (measure ADC values)	108 (36.7%)
DCE-PWI	118 (40.1%)
ASL-PWI	5 (1.7%)

QlDWI Qualitative diffusion-weighted imaging

QnDWI Quantitative diffusion-weighted imaging

DCE-PWI Dynamic contrast-enhanced perfusion-weighted imaging

ASL-PWI Arterial spin labelled perfusion-weighted imaging

Head and neck PWI was performed with arterial spin labelling (ASL) alone by 5 respondents in 4/256 centres. DCE-PWI was acquired in 135 of the remaining 252 institutions (53.6%; 95% CI: 47.2–59.9%). Routine acquisition of DCE-PWI was far less frequent than DWI, being performed for a wide range of indications in only 33/252 (13.1%; 95% CI: 9.2–17.9%) and for few specific indications in 20/252 (7.9%; 95% CI: 4.9–12.0%) (Fig. 3). Excluding the 5 respondents who analysed ASL-PWI alone, there were 118 of 289 (40.8%; 95% CI: 35.1–46.7%) respondents utilising DCE-PWI in the head and neck (Table 1 and Supplementary Fig. S3).

### Clinical indications and anatomical sites where QlDWI, QnDWI and DCE-PWI are applied

**Fig. 4:**
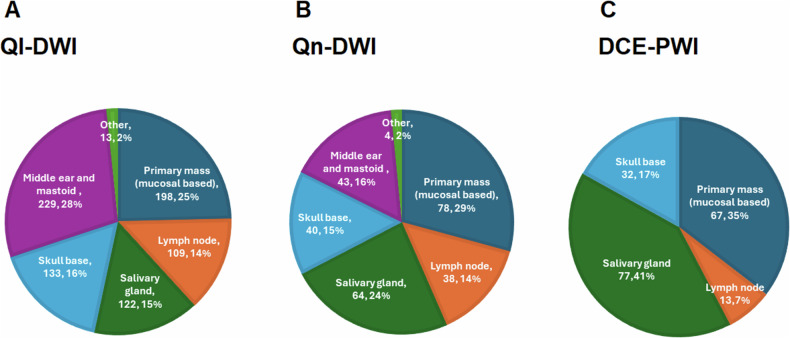
Pie charts illustrating the anatomic sites at which (**A**) QlDWI (**B**) QnDWI, and (**C**) DCE-PWI is felt most useful by respondents. Multiple responses were possible and the number of responses to each option with the relative percentages are indicated. The anatomic site as a percentage of the total respondents is indicated in the text

Ql-DWI was routinely interpreted in 3.28 ± 1.12 (*n* = 272) diagnostic clinical scenarios. The most frequent routine use of QlDWI was to diagnose cholesteatoma, as was selected by 242 of 272 users (89.0%; 95% CI: 84.6–92.4%), whilst distinguishing malignant from benign disease (233/272; 85.7%; 95% CI: 80.9–89.6%) and diagnosis of purulent fluid (213/272; 78.3%; 95% CI: 72.9–83.1%) were also frequent applications (Table 2 and Supplementary Fig. S4). The primary mucosal-based mass (198/272; 72.7%; 95% CI: 68.8–76.6%) or the middle ear and mastoid (229/272; 84.2%; 95% CI: 80.3–88.1%) were felt to be the most useful sites to apply QlDWI (Fig. 4).

**Table 2:** Clinical scenarios where respondents interpret QlDWI and analyse QnDWI

	QlDWI			QnDWI		
Diagnosis						
		% of respondents to question *n* = 272	% of total respondents *n* = 294		% of respondents to question *n* = 108	% of total respondents *n* = 294
Sometimes	22	8.1%	7.5%	28	25.9%	9.5%
Routinely to distinguish purulent (abscess) from sterile fluid	213	78.3%	**72.4%**	34	31.5%	11.6%
Routinely to distinguish and delineate malignant from benign tissue	233	85.7%	**79.3%**	63	58.3%	21.4%
Routinely to distinguish different types of tumour histology (eg lymphoma from SCC)	185	68.0%	**62.9%**	81	75.0%	27.6%
Routinely to diagnose cholesteatoma	242	89.0%	**82.3%**	39	36.1%	13.3%
Other	20	7.3%	6.8%	3	2.8%	1.0%

Treatment response		% of respondents to question *n* = 261	% of total respondents *n* = 294		% of respondents to question *n* = 99	% of total respondents *n* = 294
Sometimes	90	34.5%	30.6%	41	41.4%	13.9%
Routinely at diagnosis to predict treatment response in HN carcinoma	89	34.1%	30.3%	34	34.3%	11.6%
Routinely to determine early treatment response in HN carcinoma (< 12 weeks)	72	27.6%	24.5%	28	28.3%	9.5%
Routinely to distinguish recurrent tumours from benign post-treatment change in HN carcinoma	176	67.4%	**59.9%**	55	55.6%	18.7%
Routinely to determine treatment response in other tumours	112	42.9%	38.1%	38	38.4%	12.9%
Other	10	3.8%	3.4%	1	1.0%	0.3%

Percentages are expressed as a proportion of the total respondents to each question

Choices were divided into diagnostic scenarios and those aimed at determining treatment response. “Sometimes” indicated non-routine or occasional application

Those options which were selected by > 50% of total respondents are highlighted in bold

QlDWI Qualitative diffusion-weighted imaging

QnDWI Quantitative diffusion-weighted imaging

QnDWI was routinely applied in 2.03 ± 1.28 (*n* = 108) diagnostic clinical scenarios. It was most frequently applied to distinguish different types of tumour histology pre-treatment (81/108; 75.0%; 95% CI: 65.7–82.8%) and to distinguish recurrent tumour from benign treatment change (55/99; 55.6%; 95% CI: 45.2–65.5%) in response assessment (Table 2 and Supplementary Fig. S4). A primary mucosal-based mass was deemed the most useful site to analyse QnDWI and was selected by 78/106 (73.6%; 95% CI: 64.1–81.7%) QnDWI users. Fewer respondents selected the middle ear and mastoid (43/106; 40.6%; 95% CI: 31.1–50.5%) whilst a greater proportion selected the salivary gland 64/106 (60.4%; 95% CI: 50.4–69.7%) as compared to QlDWI (Fig. 4).

DCE-PWI was never routinely applied by more than 50% of respondents in any diagnostic clinical scenario. The most reported routine clinical indication was to distinguish and delineate malignant from benign tissue and this was selected by 50 of 118 users (42.4%; 95% CI: 33.3–51.8%) (Table 3 and Supplementary Fig. S5). Like QnDWI, the most popular anatomical sites to apply DCE-PWI were the salivary gland in 77 of 117 respondents (65.8%; 95% CI: 56.5–74.3%) and the primary mucosal-based mass in 67 of 117 respondents (57.3%; 95% CI: 47.8–66.4%) (Fig. 4). The decision to perform DCE-PWI was made by a board-certified radiologist who initially protocolled the study in 81/100 institutions (81.0%; 95% CI: 71.9–88.2%) and it was rare for it to be specifically requested by the referrer (7/100; 7.0%; 95% CI: 2.9–13.9%) (Table 4 and Supplementary Fig. S6).

**Table 3:** Clinical scenarios where respondents use DCE-PWI

	DCE-PWI	
Diagnosis	*n* = 118	
Sometimes	52	44.1%
Routinely to distinguish and delineate malignant from benign tissue	50	42.4%
Routinely to distinguish different types of tumour histology (e.g. paraganglioma/schwannoma)	41	34.7%
Other	12	10.7%
Treatment response	*n* = 118	
Sometimes	63	**53.4%**
Routinely at diagnosis to predict treatment response in HN carcinoma	16	13.6%
Routinely to determine early treatment response in HN carcinoma (< 12 weeks)	18	15.3%
Routinely to distinguish recurrent tumours from benign post-treatment change in HN carcinoma	31	26.3%
Routinely to determine treatment response in other tumours	25	21.2%
Other	7	5.9%

Percentages are expressed as a proportion of the total respondents to each question

Choices were divided into diagnostic scenarios and those aimed at determining treatment response. “Sometimes” indicated non-routine or occasional application

Those options which were selected by > 50% of respondents are highlighted in bold

DCE-PWI Dynamic contrast-enhanced perfusion-weighted imaging

**Table 4:** Person who decides whether dynamic contrast-enhanced perfusion-weighted imaging (DCE-PWI) is performed at each institution (with multiple responses permitted)

	*n* = 100	
Board-certified radiologist who “protocols” or “vets” the initial study	97	97.0%
Radiologist who recalls the patient for a second study	21	21.0%
Trainee (pre-board certification) radiologist who “protocols” or “vets” the initial study	12	12.0%
Referrer	7	7.0%
Radiographer/technologist who “protocols” or “vets” the initial study	7	7.0%
Other	3	3.0%

Percentages are expressed as a proportion of the total institutional responses to the question (*n* = 100)

### Analysis and reporting of quantitative DWI (QnDWI) and DCE-PWI

**Fig. 5:**
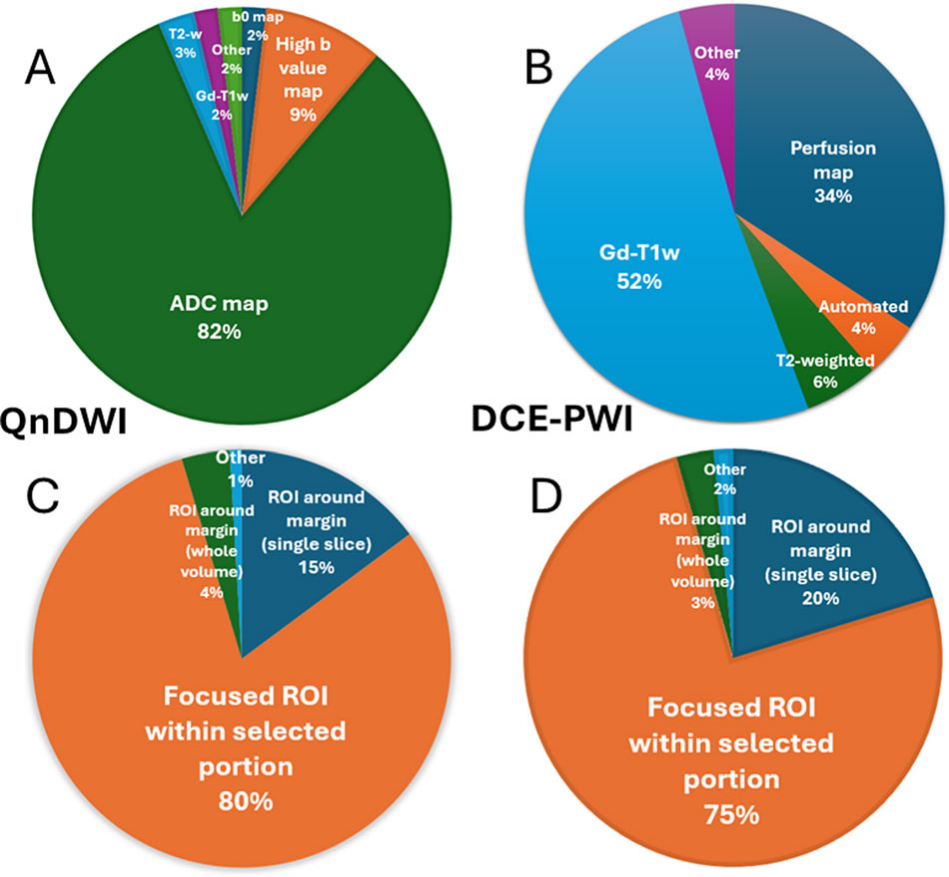
Pie charts demonstrating the sequences primarily used to draw ROIs for the analysis of (**A**) Qn-DWI (*n* = 108) and (**B**) DCE-PWI (*n* = 70) and the methods of ROI placement for (**C**) QnDWI (*n* = 108) and (**D**) DCE-PWI (*n* = 70). The percentage of total responses to the question is indicated

A QnDWI region of interest (ROI) was most frequently placed directly on the ADC map (89/108; 82.4%; 95% CI: 73.9–89.1%) whilst analysis of DCE-PWI was performed with an ROI applied to a gadolinium-enhanced image (36/70; 48.6%; 95% CI: 36.4–60.8%) or a perfusion map (24/70; 34.3%; 95% CI: 22.3–46.7%) (Fig. 5). The radiologist most frequently placed a focused ROI within a selection of the lesion for both QnDWI (87/108; 80.6%; 95% CI: 71.8–87.5%) and DCE-PWI (57/70; 75.4%; 95% CI: 63.5–84.9%) (Fig. 5). Necrosis was “always avoided” when analysing QnDWI by 97/108 respondents (89.8%; 95% CI: 82.5–94.8%) and when analysing DCE-PWI by 63/69 respondents (91.3%; 95% CI: 82.0–96.7%) but was at least “sometimes avoided” by all respondents.

Most radiologists using DCE-PWI analysed either semi-quantitative (time-intensity curves) or kinetic quantitative parameters (70/108; 59.3%; 95% CI: 49.9–68.3%) with the remainder performing qualitative analysis alone. Most of these radiologists analysing DCE-PWI reported curve pattern analyses of the time-intensity curves (TIC) (57/70; 81.4%; 95% CI: 70.3–89.7%). Kinetic quantitative DCE-PWI parameters were only reported by 9 of 70 radiologists analysing DCE-PWI (12.9%; 95% CI: 6.1–23.0%) which represented 3.1% (95% CI: 1.4–5.7%) of overall respondents.

Post-processing within institutions performing DCE-MRI was most frequently undertaken by the radiologist using third-party software (35/62; 56.5%; 95% CI: 43.3–69.0%) or directly within PACS (13/62; 21.0%; 95% CI: 11.7–33.2%). It was rarely performed by radiographers or technical support (11/62; 17.7%; 95% CI: 9.2–29.5%) (Table 5, Supplementary Fig. S7, and Supplementary Table S2).

**Table 5:** Procedure for post-processing and creation of perfusion maps or time-intensity curves for DCE-PWI by institution (with multiple responses permitted)

	*n* = 62	
By radiologists using third-party processing software and sending it to PACS	38	60.3%
By radiologists directly within PACS	17	27.4%
By radiographers or technical support and made available to radiologists on PACS	12	19.4%
Using third-party automated post-processing software	3	4.8%

Percentages are expressed as a proportion of the total institutional responses to the question (*n* = 62)

### Barriers to implementation and future intentions to use qDWI and DCE-PWI

**Fig. 6:**
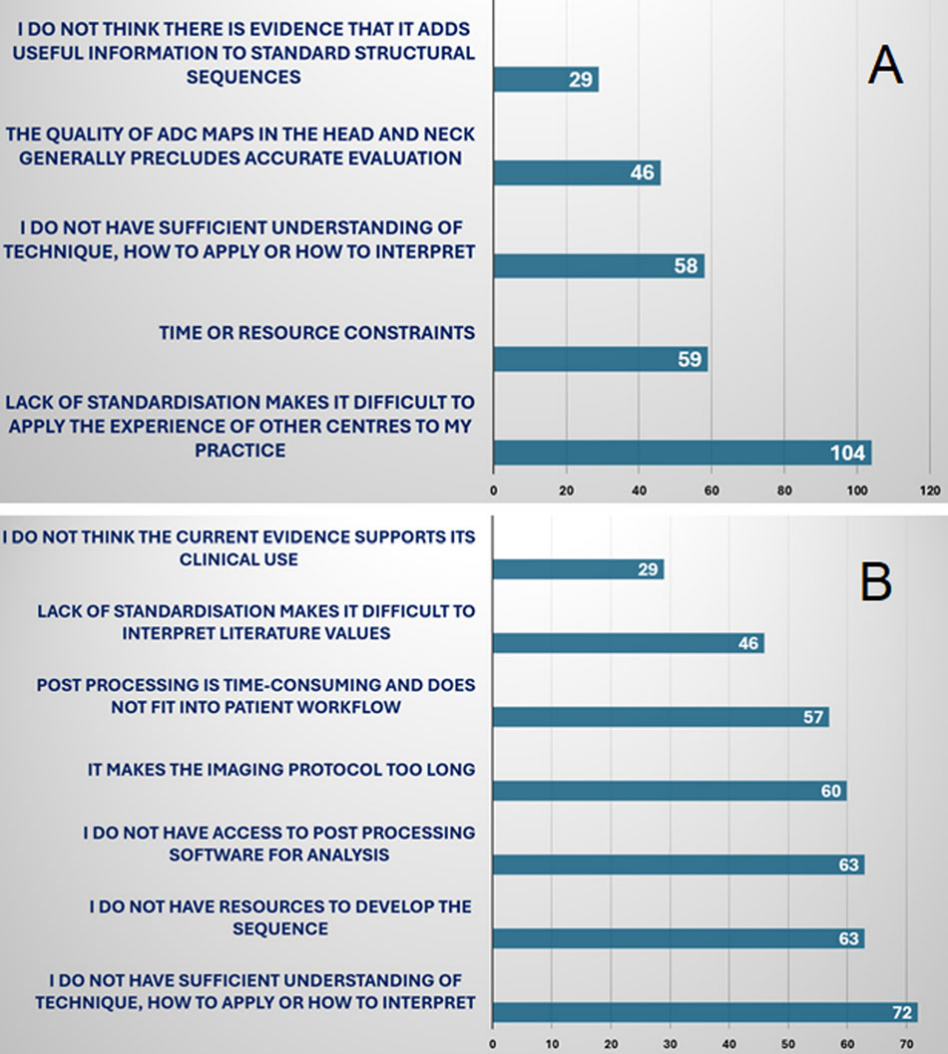
Bar charts demonstrating the reasons why QnDWI and DCE-PWI are not implemented. Respondents were asked why they or their institutions did (**A**) not analyse (measure) ADC (*n* = 186) or **B** not use DCE-PWI (*n* = 176). The number of responses to each barrier is indicated and multiple responses were permitted

The most frequent explanation for not analysing ADC value was that the lack of standardisation made it difficult to apply (104/186; 44.1%; 95% CI: 36.8–51.5%). There were found to be multiple barriers preventing the adoption of DCE PWI with 2.32 + −1.48 (*n* = 176) reasons provided by each respondent. Insufficient understanding of how to apply the technique was the most frequent concern (72/176; 40.9%; 95% CI: 33.6–48.6%) (Fig. 6).

Most radiologists who were not currently applying QnDWI indicated that they would like to start using it in the future (153/172; 89.0%; 95% CI: 83.3–93.2%). However, most (111/153; 89.0%; 95% CI: 83.3–93.2%) highlighted that they would require more resources, education and/or technical help for this to be feasible. Similarly, whilst there was interest in developing DCE-PWI (145/176; 82.4%; 95% CI: 75.9–87.7%), it was highlighted this would require support (108/145; 89.0%; 95% CI: 83.3–93.2%) in most cases.

### Group comparisons

Head and neck DWI was more likely to be routinely acquired in private practice than public or academic institutions (Chi sq 3.955, *p* = 0.047), however routine institutional acquisition of DWI in the head and neck was not significantly associated with the number of radiologists reporting head and neck MRI, or whether they were based in Europe or North America. The sample sizes were sufficient to detect an effect size of 0.2 with a power of > 80%.

Head and neck DCE-PWI was more likely to be routinely acquired in private practice than public or academic institutions (Chi sq 5.156, *p* = 0.023) but was also more frequently acquired by European institutions than those in North America (Chi sq 11.723, *p* < 0.001) and when there were > 7 radiologists reporting head and neck MRI (Chi sq 5.156, *p* = 0.023).

There was no significant difference between the proportions of European and North American radiologists utilising QlDWI, QnDWI, or DCE-PWI. The sample size was sufficient to detect an effect size of 0.2 with a power of > 80%.

## Discussion

This international survey was conducted to provide insight into the current practice of DWI and DCE-PWI in the head and neck, exploring the utilisation, most frequent clinical applications, methods of analysis and barriers to applying these techniques. The survey response rate of 17.6% compares well with similar questionnaires [36–42]. Head and neck DWI was routinely acquired at > 95% of institutions, with radiologists usually interpreting QlDWI (92.5%) but only a minority analysed QnDWI (36.7%). Head and neck DCE-PWI was acquired at 53.6% of institutions and utilised by 40.8% of respondents but only routinely applied in 21%, with most radiologists performing either qualitative or semi-quantitative analysis. QlDWI was routinely interpreted across a broad range of clinical indications and most frequently applied to the primary mucosal masses or middle ear pathologies. QnDWI was most frequently routinely used to distinguish tumour histologies and primary or recurrent carcinoma. There was no clinical indication for DCE-PWI which was routinely applied by most users. DCE-PWI analysis methods varied, with time-intensity curve classifications being the most frequently reported. Lack of standardisation across centres was a key reason for not implementing QnDWI, whilst a wide range of factors impeded the adoption of DCE-PWI. There was considerable interest in initiating these techniques, representing an endorsement of their potential benefits by head and neck radiologists.

Whilst this represents the first survey dedicated to DWI and PWI in the head and neck, previous surveys have addressed PWI within the wider neuroradiological setting. Manfrini et al reported in 2021 that DCE-PWI was applied in the head and neck by 20.6% of 272 European neuroradiologists, and that lesion differentiation was the most frequent indication for quantitative PWI techniques [40]. The survey by Dickerson et al in 2016 found that 5.1% of 158 USA institutions acquiring PWI were applying it to the primary evaluation of head and neck cancer [36]. No previous surveys have specifically addressed the use of DWI in the head and neck.

Our survey responses demonstrate a limited translation of QnDWI and DCE-PWI into clinical head and neck imaging when compared to QlDWI. One of the key concerns, preventing the wider application of qDWI, was the lack of standardisation such that similar methods and threshold parameters could not be applied across the scanner and vendor platforms. Our survey highlighted that lack of access to post-processing software, imaging acquisition time and analysis time was preventing approximately one-third of radiologists from utilising head and neck DCE-PWI. This is a particular challenge as post-COVID imaging backlogs, radiology workforce shortages and limited healthcare budgets [43] are current drivers for shortened MRI protocols, reduced reporting turnaround and decreased investment in new infrastructure. Insufficient understanding of the technique was also proposed as a frequent barrier to adopting DCE-PWI and education emerged as a key requirement for those who aspired to apply DCE-MRI. Interestingly, the lack of evidence for its clinical use was less frequently emphasised, although undoubtedly additional research would support guidelines and justify funding of health care resources [44]. The variation in the acquisition of both DWI and DCE-PWI with geography, practice settings and department size confirms a need to equalise access of radiologists and patients to these techniques.

There were similarities between the most frequent methods of ROI placement for the quantification of DWI and DCE-PWI, with focused ROIs usually placed to avoid necrosis. Despite data from the whole tumour volume being more representative [13], this was rarely applied. Interestingly, there were only two reports of automated ROI placement, however, it is anticipated the post-processing of this quantifiable data will become quicker and more consistent with the use of such software packages in the future. Moreover, an understanding of the application and limitations of QnDWI and DCE-PWI will become more important in the future, as routine radiomics allows the extraction of innumerable quantitative features.

There are limitations to the results of this survey which should be addressed. Firstly, there is potential bias due to the questionnaire being confined to ASHNR and ESHNR members, as it may be hypothesised that society members are more likely to have an inherent interest in developing such imaging techniques. Secondly, the low (17.6%) overall response rate, and the imbalance in responses between the two societies, represent a major flaw of the study. Due to the introduction of selection bias, the answers of the minority of head and neck radiologists who responded may not be representative of all those surveyed. For instance, radiologists may have been more motivated to complete the survey if they were practising these MRI techniques, and an uneven response according to geographic and practice setting may have distorted the survey outcomes. Thirdly, the survey was not comprehensive, as for example pertinent questions on technical aspects of image acquisition and analysis could not be included within the proposed 5-min completion time. Finally, although data was presented only in aggregate, the request for contact details could have influenced outcomes.

In conclusion, this international survey of DWI and DCE-PWI in the head and neck highlights the widespread interpretation of QlDWI by head and neck radiologists, but more limited translation of quantitative advanced MRI techniques into the clinical environment. The survey demonstrates considerable variations in the utilisation, clinical application and analysis of these techniques. Whilst the head and neck imaging community demonstrates a strong interest in developing QnDWI and DCE-PWI, there are several perceived barriers to overcome. Although the low response rate may introduce selection bias and influence the survey outcomes, it is hoped the results will aid discussion on how to standardise and disseminate optimal practice guidelines for advanced head and neck MRI and how to justify the provision of targeted education and resources to health care organisations.

## Supplementary information

ELECTRONIC SUPPLEMENTARY MATERIAL

## Abbreviations

ASHNR: American Society of Head and Neck Radiology
DWI: Diffusion-weighted imaging
DCE-PWI: Dynamic contrast-enhanced perfusion-weighted imaging
ESHNR: European Society of Head and Neck Radiology
PWI: Perfusion-weighted imaging
QlDWI: Qualitative DWI
QnDWI: Quantitative DWI
