# Supplementary material for: An international survey of diffusion and perfusion magnetic resonance imaging implementation in the head and neck
Source: Eur Radiol. 2025 Feb 4;35(8):5110–20. doi: 10.1007/s00330-025-11370-1 (PMC12226617; doi:10.1007/s00330-025-11370-1)
Supplement: Supplementary file 1 — ELECTRONIC SUPPLEMENTARY MATERIAL [file 330_2025_11370_MOESM1_ESM.pdf]

An international survey of diffusion and perfusion magnetic resonance imaging implementation in the head and neck

ELECTRONIC SUPPLEMENTARY MATERIAL

Eur Radiol (2025) Connor S, Christoforou A, Touska P, et al.

Supplementary figure S1: Survey questions. Note that question 1 also refers to membership of the ASNR, however the questionnaire was not distributed to members of the ASNR alone.

Thank you for participating in this international survey of current practice in diffusion and perfusion imaging of the head and neck.

This survey is formulated by representatives from both the European and the American Societies of Head and Neck Radiology.

- We have focused on questions which help us to better understand when and how these techniques are being practiced by head and neck radiologists worldwide, and whether there are any barriers to their implementation.

- The results from this survey will be made available and will help head and neck radiologists standardise practice whilst also providing pertinent information to health care organisations and funders.

- It will usually require less than 5 minutes to complete the survey and we are grateful for your precious time.

- None of your identifying information (including institution/hospital) will be shared.

- **You may leave out questions if you are uncertain of the answer (questions you must complete will be indicated by a *).**

* Required

I have received this survey from *

Select one answer

- ◯ ESHNR
- ◯ ASNR/ASHNR

What is the name of your main institution? *

(so we may identify duplicate responses from the same hospital or institution)

For this survey, we are using the term institution to represent clinics, hospitals, institutions, etc.

Please provide hospital name and city

Would you describe your main/primary practice setting as? *

Select one answer (the best option)
Subsequent responses will pertain to this practice setting

- ◯ Academic
- ◯ Public
- ◯ Private
- ◯ Other

How many attending/consultant/post certification radiologists regularly report MRI of the head and neck? (at your main/primary practice) *

Select one answer

- ◯ 0-3
- ◯ 4-7
- ◯ >7

Does YOUR INSTITUTION acquire DWI MRI in the head and neck? *

Select one answer

- ◯ Never
- ◯ Occasionally at our discretion in a few specific indications
- ◯ Routinely for a few specific indications
- ◯ Routinely for a wide range of indications

Do YOU qualitatively interpret DWI MRI in the head and neck? *

- ◯ I do interpret DWI signal and/or ADC maps
- ◯ I do not interpret DWI signal and/or ADC maps

I interpret DWI signal and/or ADC maps:

select any/all that apply

Diagnosis

☐ a) sometimes

☐ b) routinely to distinguish purulent (abscess) from sterile fluid

☐ c) routinely to distinguish and delineate malignant from benign tissue

☐ d) routinely to distinguish different types of tumour histology (eg lymphoma from SCC)

☐ e) routinely to diagnose cholesteatoma

☐ Other

Treatment response

☐ f) sometimes

☐ g) routinely at diagnosis to predict treatment response in HN carcinoma

☐ h) routinely to determine early treatment response in HN carcinoma (<12 weeks)

☐ i) routinely to distinguish recurrent tumour from benign post treatment change in HN carcinoma

☐ j) routinely to determine treatment response in other tumours

☐ Other

At which sites do YOU find it most useful

select any/all that apply

☐ Primary mass (mucosal based)

☐ Lymph node

☐ Salivary gland

☐ Skull base

☐ Middle ear and mastoid

☐ Other

Do YOU quantitatively analyse (measure) ADC values in the head and neck? *

◯ I do quantitatively analyse (measure) ADC signal

◯ I do not quantitatively analyse (measure) ADC signal

Why do you or your institution not analyse (measure) ADC signal in the head and neck? *

select any/all that apply

☐ a) I do not think there is evidence that it adds useful information to standard structural sequences

☐ b) lack of standardisation makes it difficult to apply the experience of other centres to my practice

☐ c) I do not have sufficient understanding of technique, how to apply or how to interpret

☐ d) the quality of ADC maps in the head and neck generally precludes accurate evaluation

☐ e) time or resource constraints

☐ Other

Would you like to start using quantitative diffusion head and neck imaging in the future? *

◯ Yes

◯ Yes, but only if provided with further resources/education/technical support

◯ No

I measure ADC values:

select any/all that apply

Diagnosis

- [ ] a) sometimes
- [ ] b) routinely to distinguish purulent (abscess) from sterile fluid
- [ ] c) routinely to distinguish and delineate malignant from benign tissue
- [ ] d) routinely to distinguish different types of tumour histology (eg lymphoma from SCC)
- [ ] e) routinely to diagnose cholesteatoma
- [ ] Other

Treatment response

- [ ] f) sometimes
- [ ] g) routinely at diagnosis to predict treatment response in HN carcinoma
- [ ] h) routinely to determine early treatment response in HN carcinoma (<12 weeks)
- [ ] i) routinely to distinguish recurrent tumour from benign post treatment change in HN carcinoma
- [ ] j) routinely to determine treatment response in other tumours
- [ ] Other

At which sites do YOU find it most useful

select any/all that apply

- [ ] a) Primary mass (mucosal based)
- [ ] b) Lymph node
- [ ] c) Salivary gland
- [ ] d) Skull base
- [ ] e) Middle ear and mastoid
- [ ] Other

Which sequence do you primarily use to draw ROI?

Select one answer

- ◯ B0 or low B value map
- ◯ High b value DWI map
- ◯ ADC map
- ◯ T2-weighted
- ◯ Gadolinium enhanced T1w
- ◯ Automated
- ◯ Other

How are regions of interest usually placed to measure ADC?

Select one answer (the best option)

- ◯ Freehand ROI around the margin outlining whole lesion (on a single slice)
- ◯ Focused ROI within a selected portion of the lesion (on a single slice)
- ◯ Freehand ROI around on all sections of entire lesion (whole volume)
- ◯ Other

Do you avoid areas of 'necrosis/cystic change' when drawing your ROI?

- ◯ Yes
- ◯ No
- ◯ Sometimes

Does YOUR INSTITUTION acquire dynamic contrast enhanced (DCE) perfusion MR imaging in the head and neck? *

- ◯ Never
- ◯ Occasionally at our discretion in a few specific indications
- ◯ Always and routinely for a few specific indications
- ◯ Always and routinely for a wide range of indications
- ◯ We acquire only arterial spin labelling arterial spin labelling (ASL) perfusion imaging

Why do you not use DCE MR perfusion imaging in the head and neck? *

select any/all that apply

- ☐ I do not think the current evidence supports its clinical use
- ☐ Lack of standardisation makes it difficult to interpret literature values
- ☐ I do not have sufficient understanding of technique, how to apply or how to interpret
- ☐ I do not have resources to develop the sequence
- ☐ It makes the imaging protocol too long
- ☐ I do not have access to post processing software for analysis.
- ☐ Post processing is time-consuming and does not fit into patient workflow
- ☐ Other

Would you like to start using quantitative perfusion head and neck imaging in the future? *

- ◯ Yes
- ◯ Yes, but only if provided with further resources/education/technical support
- ◯ No

Do YOU use DCE perfusion MR imaging *

- ◯ I do use DCE perfusion MR imaging
- ◯ I do not use DCE perfusion MR imaging

Why do you not use DCE MR perfusion imaging in the head and neck? *

select any/all that apply

- ☐ I do not think the current evidence supports its clinical use
- ☐ Lack of standardisation makes it difficult to interpret literature values
- ☐ I do not have sufficient understanding of technique, how to apply or how to interpret
- ☐ I do not have resources to develop the sequence
- ☐ It makes the imaging protocol too long
- ☐ I do not have access to post processing software for analysis.
- ☐ Post processing is time-consuming and does not fit into patient workflow

Would you like to start using quantitative perfusion head and neck imaging in the future? *

- ◯ Yes
- ◯ Yes, but only if provided with further resources/education/technical support
- ◯ No

I use MR DCE perfusion imaging:

select any/all that apply

Diagnosis

- [ ] a) sometimes
- [ ] b) routinely to distinguish and delineate malignant from benign tissue
- [ ] c) routinely to distinguish different types of tumour histology (e.g. paraganglioma/ schwannoma)
- [ ] Other

Treatment response

- [ ] d) sometimes
- [ ] e) routinely at diagnosis to predict treatment response in HN carcinoma
- [ ] f) routinely to determine early treatment response in HN carcinoma (<12 weeks)
- [ ] g) routinely to distinguish recurrent tumour from benign post treatment change in HN carcinoma
- [ ] h) routinely to determine treatment response in other tumours
- [ ] Other

At which sites do YOU find it most useful

select any/all that apply

- [ ] Primary mass (mucosal based)
- [ ] Lymph node
- [ ] Salivary gland
- [ ] Skull base
- [ ] Other

Who decides when DCE perfusion MRI will be performed at your institution?

select any/all that apply

- ☐ a referrer
- ☐ a trainee (pre board certification) radiologist who "protocols" or "vets" the initial study
- ☐ a board-certified radiologist who "protocols" or "vets" the initial study
- ☐ a radiographer/technologist who "protocols" or "vets" the initial study
- ☐ a radiologist who recalls the patient for a second "recall" study
- ☐ Other

I perform semi-quantitative (time intensity curves) or quantitative analysis (pharmokinetic models with parameters such as Ktrans) on the DCE perfusion MR imaging *

- ○ Yes
- ○ No (I only perform qualitative analysis)

Would you like to start using quantitative perfusion head and neck imaging in the future? *

- ○ Yes
- ○ Yes, but only if provided with further resources/education/technical support
- ○ No

Which sequence do you primarily use to draw ROI?

Select one answer

- ◯ T2-weighted
- ◯ Gadolinium enhanced T1-weighted
- ◯ Perfusion maps
- ◯ Automated
- ◯ Other

How are regions of interest usually placed?

Select one answer (the best option)

- ◯ Freehand ROI around the margin outlining whole lesion (on a single slice)
- ◯ Focused ROI within a selected portion of the lesion (on a single slice)
- ◯ Freehand ROI around on all sections of entire lesion (whole volume)
- ◯ Other

Do you avoid areas of 'necrosis/cystic change' when drawing your ROI?

- ◯ Yes
- ◯ No
- ◯ Sometimes

Regarding post processing and creation of the perfusion maps or time intensity curves

This is performed:
select one answer

- ◯ a) by radiographers or technical support and made available to radiologist on PACS
- ◯ b) by radiologists directly within PACS
- ◯ c) by radiologists using third party post processing software and sent to PACS
- ◯ d) using third party automated post processing software and sent to PACS
- ◯ Other

which vendor?

Regarding reporting of DCE MR perfusion parameters
select any/all that apply (more than one option possible)

- ☐ parameters are not added to the report (I only use them to help me interpret)
- ☐ I report curve pattern classifications from time intensity curve
- ☐ I report semi quantitative parameters from time intensity curve (eg maximum slope, AUC)
- ☐ I report kinetic quantitative parameters such as K trans or other constants
- ☐ I report specific threshold values used to interpret MR perfusion parameters

Thank you for completing the survey

Please supply email address (it will not be shared) *

Supplementary figure S2: Map of Europe indicating the number of respondents from each country.

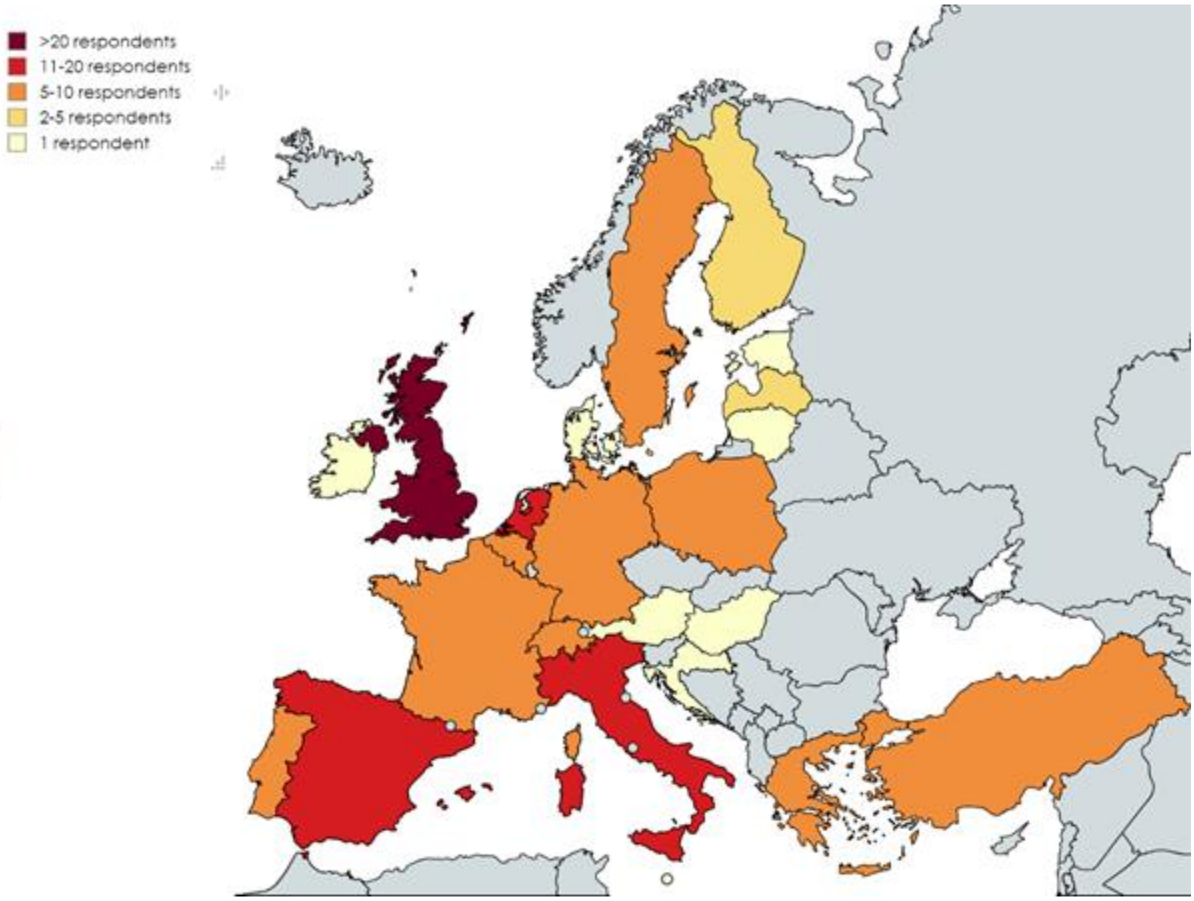

Supplementary figure S3: Bar chart indicating the number of respondents interpreting QlDWI, analysing QnDWI and using DCE-PWI (n=294). Note the 5 respondents using ASL-PWI alone are excluded from the DCE-PWI bar.

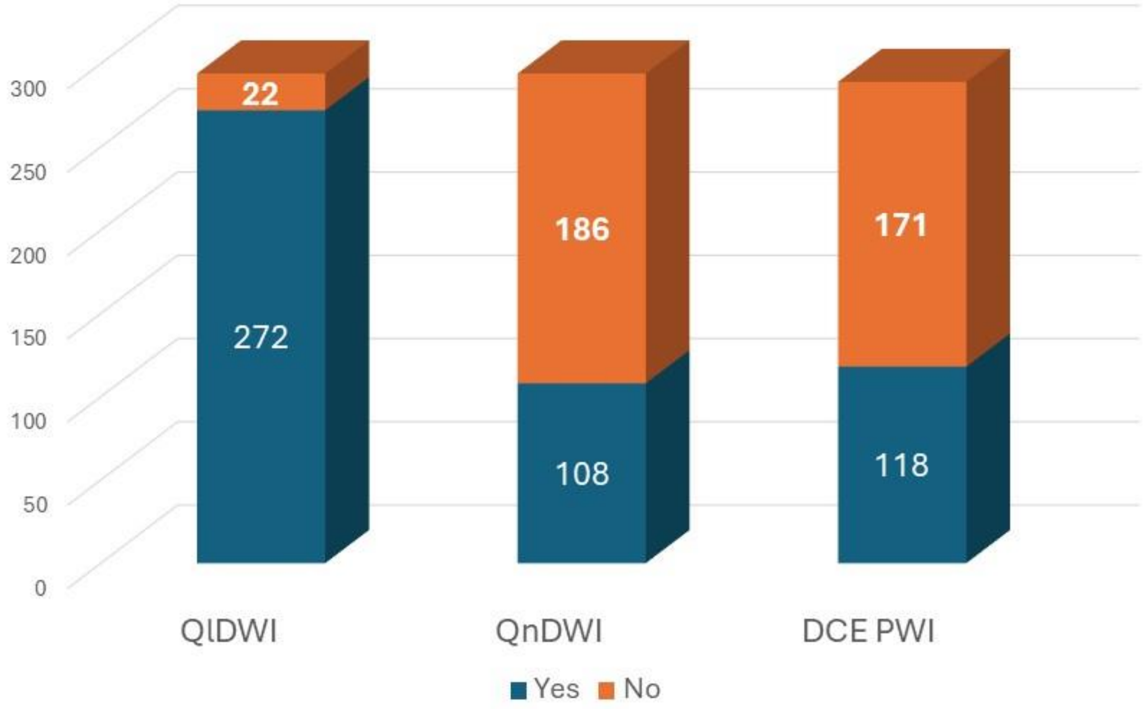

Supplementary figure S4: Cluster bar chart demonstrating the clinical scenarios in which QlDWI and QnDWI are applied in the head and neck. The relative number of responses for each pre-treatment diagnostic application are on the left of each cluster (QlDWI n=272, QnDWI n=108) and post-treatment applications are on the right (QlDWI n=261, QnDWI n=99).

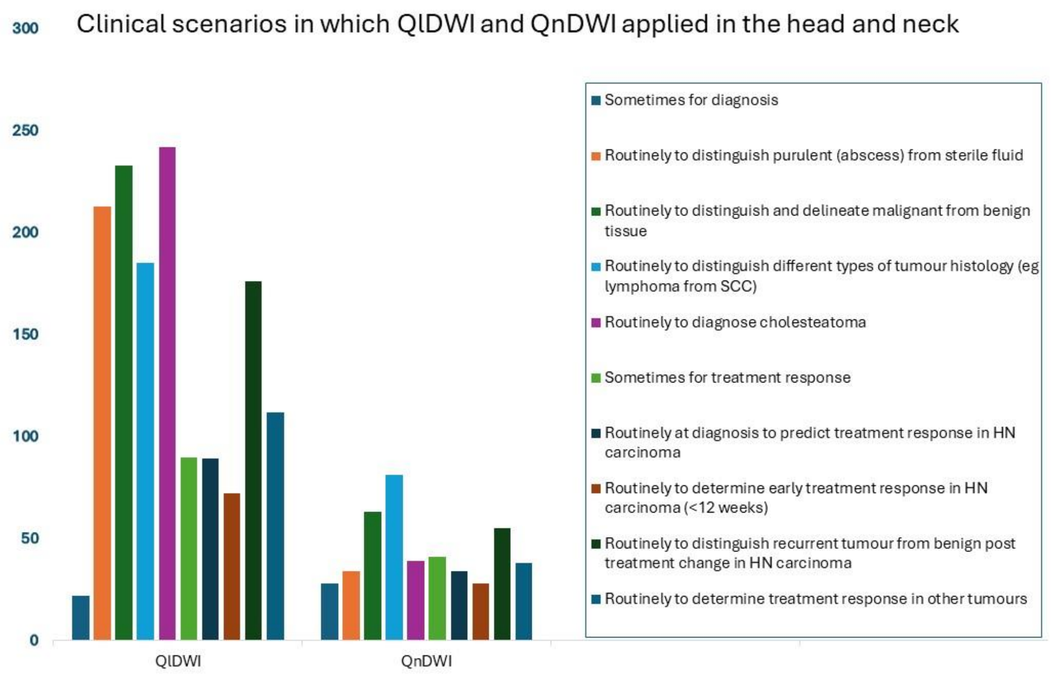

Supplementary figure S5: Cluster bar chart demonstrating the clinical scenarios in which DCE-DWI is applied in the head and neck. The relative number of responses for each pre-treatment diagnostic application are on the left and post-treatment applications are on the right (n=118 for both).

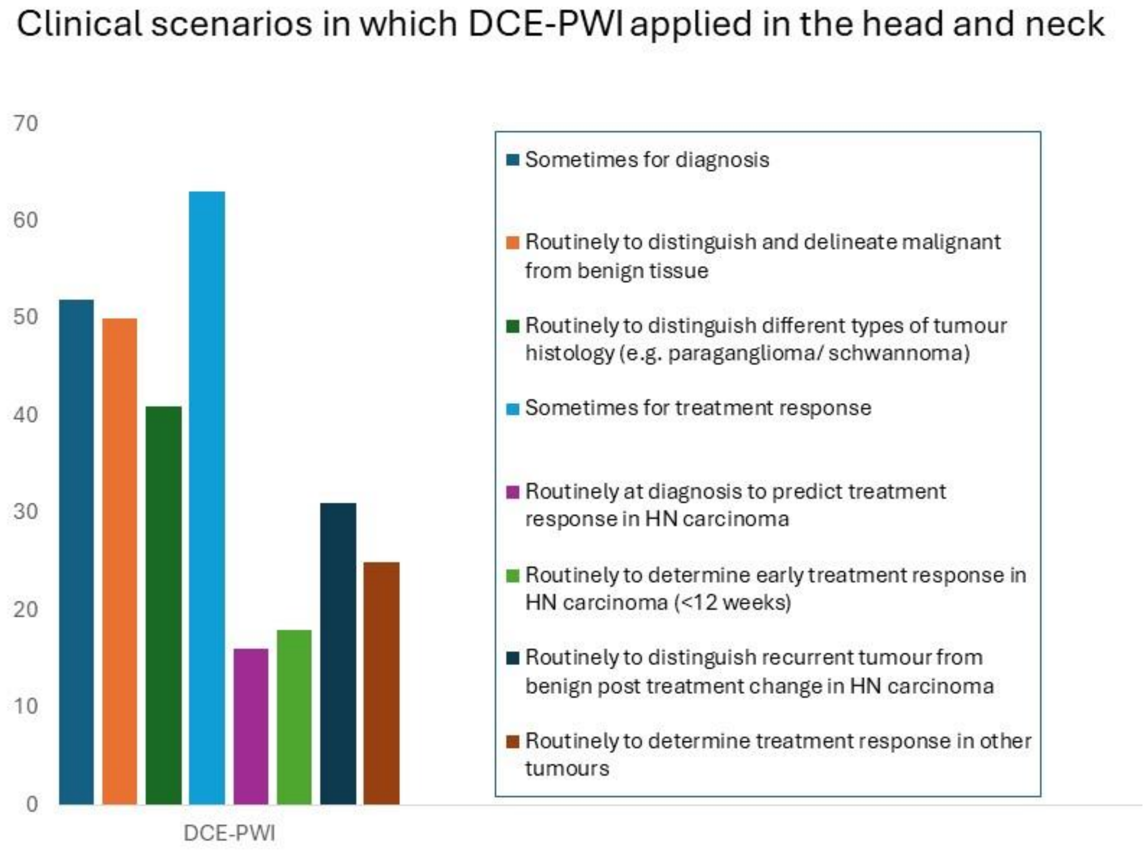

Supplementary figure S6: Bar chart indicating who decides when DCE-PWI is to be performed by institution (n=100).

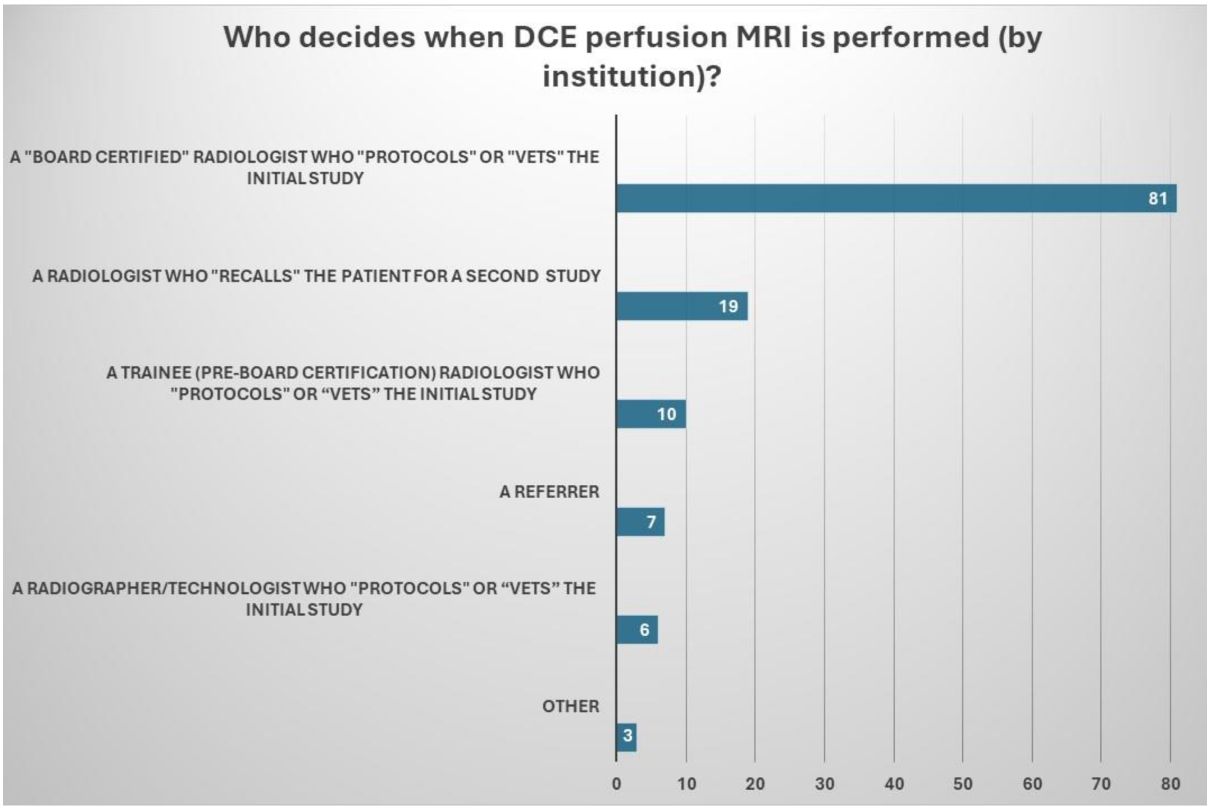

Supplementary figure S7: Bar chart demonstrating procedure for post-processing and creation of perfusion maps or time intensity curves for dynamic contrast enhanced perfusion weighted imaging (DCE-PWI) by institution (n=62).

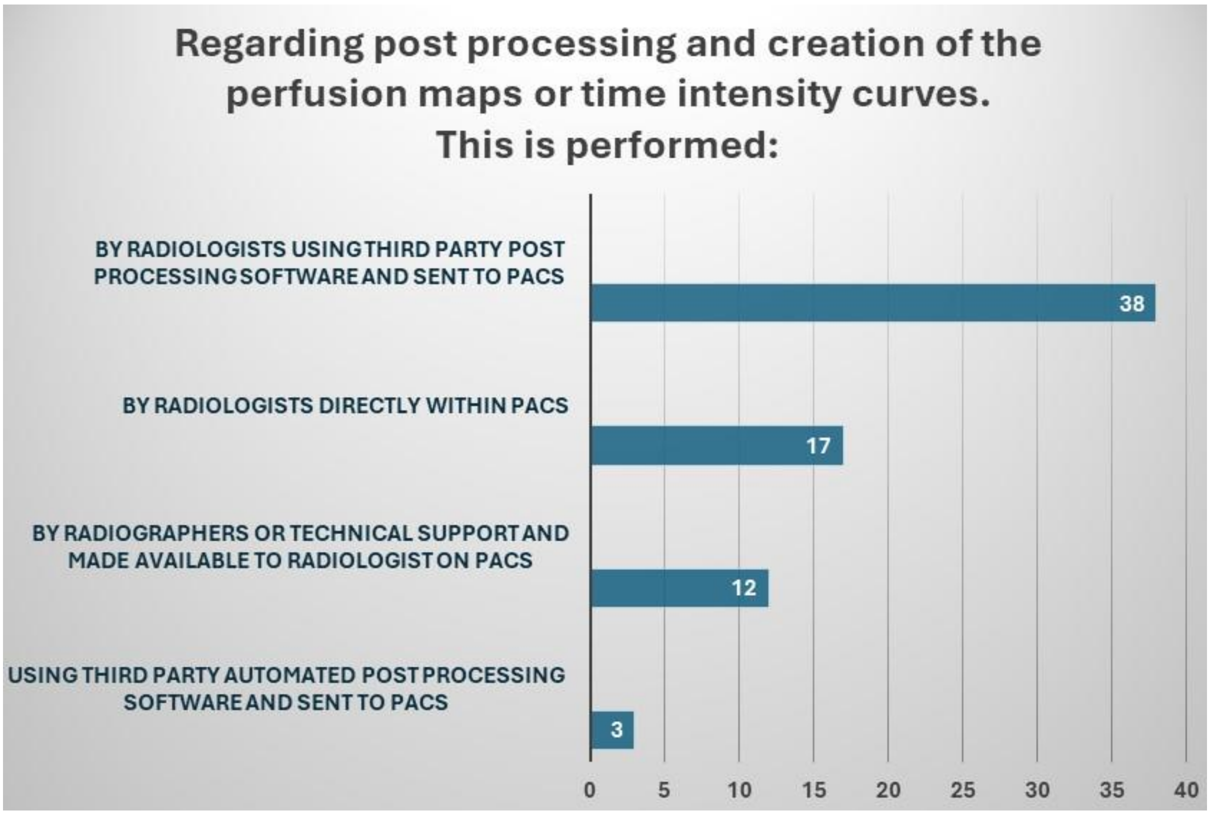

Supplementary table S1
Number of respondents from each country

**Country**	**n=294**
USA	63
United Kingdom	48
India	18
Italy	12
Netherlands	12
Spain	11
Switzerland	10
Turkey	10
Germany	9
Australia	9
France	9
Belgium	8
Sweden	8
Portugal	8
Poland	7
Greece	5
Mexico	5
Canada	3
Finland	3
Singapore	3
South Korea	2

Brazil	2
Chile	2
Malaysia	2
Nigeria	2
South Africa	2
Latvia	2
Brazil	2
Austria	1
Hungary	1
Denmark	1
Lithuania	1
Estonia	1
Malta	1
Croatia	1
Ireland	1
Ethiopia	1
Thailand	1
Egypt	1
UAE	1
Qatar	1
Uzbekistan	1
New Zealand	1
Israel	1
Hong Kong	1

Supplementary table S2: Which vendor is used to post process perfusion maps or time intensity curves?

Siemens	14
Olea	5
General Electric	5
Philips	14
